# Symptom Presentation in Idiopathic Environmental Intolerance With Attribution to Electromagnetic Fields: Evidence for a Nocebo Effect Based on Data Re-Analyzed From Two Previous Provocation Studies

**DOI:** 10.3389/fpsyg.2018.01563

**Published:** 2018-08-28

**Authors:** Stacy Eltiti, Denise Wallace, Riccardo Russo, Elaine Fox

**Affiliations:** ^1^Rosemead School of Psychology, Biola University, La Mirada, CA, United States; ^2^Department of Psychology, Centre for Brain Science, University of Essex, Colchester, United Kingdom; ^3^Department of Brain and Behavioral Sciences, University of Pavia, Pavia, Italy; ^4^Department of Experimental Psychology, University of Oxford, Oxford, United Kingdom

**Keywords:** electromagnetic hypersensitivity, idiopathic environmental illness, nocebo effect, mobile phone base station, well-being, electromagnetic fields

## Abstract

Individuals with idiopathic environmental illness with attribution to electromagnetic fields (IEI-EMF) claim they experience adverse symptoms when exposed to electromagnetic fields (EMFs) from mobile telecommunication devices. However, research has consistently reported no relationship between exposure to EMFs and symptoms in IEI-EMF individuals. The current study investigated whether presence of symptoms in IEI-EMF individuals were associated with a nocebo effect. Data from two previous double-blind provocation studies were re-analyzed based on participants’ judgments as to whether or not they believed a telecommunication base station was “on” or “off”. Experiment 1 examined data in which participants were exposed to EMFs from Global System for Mobile Communication, Universal Mobile Telecommunications System, and sham base station signals. In Experiment 2, participants were exposed to EMFs from Terrestrial Trunked Radio Telecommunications System and sham base station signals. Our measures of subjective well-being indicated IEI-EMF participants consistently reported significantly lower levels of well-being, when they believed the base station was “on” compared to “off”. Interestingly, control participants also reported experiencing more symptoms and greater symptom severity when they too believed the base station was “on” compared to “off”. Thus, a nocebo effect provides a reasonable explanation for the presence of symptoms in IEI-EMF and control participants.

## Introduction

Idiopathic environmental intolerance with attribution to electromagnetic fields (IEI-EMF), formerly electrosensitivity, is an illness comprising medically unexplained symptoms in which individuals have a strongly held belief that their symptoms are caused by exposure to electromagnetic fields (EMF) (Hillert et al., 2006). Symptoms vary from person to person, but typically include headaches, pain, or sensation of heating in the face or ear, skin irritations, and cognitive difficulties. There are also a variety of objects that these individuals believe trigger their symptoms, such as mobile phones, mobile phone base stations, WiFi, computers, and so on.

Several large-scale double-blind placebo-controlled provocation studies have examined the relationship between exposure to EMF and well-being in both IEI-EMF and control individuals (Regel et al., 2006; Rubin G. et al., 2006; Wallace et al., 2010; Nieto-Hernandez et al., 2011). These studies have found that, although IEI-EMF individuals consistently report lower levels of well-being than controls, the presence of their symptoms are not associated with short-term exposure to EMF from mobile phone technology. The consensus from several reviews of the literature is that there is no scientific evidence for a causal association between EMF exposure and symptoms experienced by either IEI-EMF or control individuals (Kwon and Hämäläinen, 2010; Röösli et al., 2010; Rubin et al., 2010, 2011). Moreover, neither controls nor IEI-EMF participants can correctly judge when they are receiving real exposure compared to sham (Eltiti et al., 2007a; Kwon and Hämäläinen, 2010; Röösli et al., 2010; Rubin et al., 2010; Wallace et al., 2010). Given this lack of evidence to support their deeply held belief, the question of what is causing the very real and often disabling IEI-EMF symptoms remains. One possibility is that the symptoms are the result of a nocebo effect. That is, the presence and maintenance of symptoms is due to a strong underlying belief that EMFs are harmful, rather than actual EMF exposure (Rubin G. et al., 2006; Stovner et al., 2008; Kwon and Hämäläinen, 2010; Rubin et al., 2010). More recently, Van den Bergh et al. (2017) have proposed that the presence of symptoms in IEI individuals are the result of two processes: (a) prior expectations amplify imprecise benign physiological inputs and (b) symptoms then become associated with environmental factors that eventually lead to a nocebo response via interoceptive conditioning.

The nocebo effect has typically been researched in terms of the impact of suggestibility on the perception of pain (Colloca et al., 2008) as well as reports of side effects from placebo–controlled drug studies (Ferguson, 1993; Colloca and Miller, 2011; Mitsikostas et al., 2011). A handful of studies have examined the nocebo effect in the context of perceived exposure to EMFs (Schweiger and Parducci, 1981; Landgrebe et al., 2008; Szemerszky et al., 2010; Witthöft and Rubin, 2013). In these studies, non-sensitive participants are led to believe that a particular symptom(s) would occur due to exposure to an EMF-producing device. Participants are then told that they are being exposed to EMFs and their symptoms are recorded. Using this methodology, Schweiger and Parducci (1981) found that the majority of participants who were led to believe that exposure to low-voltage EMF would induce a headache did indeed report experiencing a headache during *perceived* exposure to a low-voltage EMF device. Personality factors, such as high state anxiety (Witthöft and Rubin, 2013), high concern regarding EMF exposure (Witthöft and Rubin, 2013), and self-reported IEI-EMF (Szemerszky et al., 2010), have all been shown to be important in eliciting nocebo effects in response to perceived EMF exposure. Only one study involved preselected IEI-EMF individuals and compared their performance to control participants (Landgrebe et al., 2008). IEI-EMF participants showed heightened activation in the anterior cingulate cortex and anterior insula both in anticipation of and during a sham EMF mobile phone exposure. These areas are believed to be involved in both the anticipation and perception of either painful or unpleasant stimuli and the manifestation of symptoms. It is important to note that in each of these studies, participants were not actually exposed to EMF, but were all led to believe that they were being exposed. Additionally, none of these experiments included a comparison control condition in which participants reported symptoms under a supposedly no-exposure condition.

Given that IEI-EMF individuals consistently report symptoms during provocation studies with no evidence linking symptoms to actual EMF exposure, the purpose of the present study was to explore if a nocebo effect could explain the presence of symptoms in IEI-EMF individuals. To achieve this, subjective well-being data from two previous double-blind provocation studies (Eltiti et al., 2007a; Wallace et al., 2010), which were procedurally identical were extracted. In both studies, participants were exposed to sham and real EMFs from a mobile communication base station. Given that the primary interest was the impact of belief on subjective well-being, the data were examined in relation to what participants thought they were being exposed to rather than actual exposure. On each test occasion, participants were asked to judge whether or not they believed the mobile phone base station was either “on” or “off”. These judgments, therefore, served as a measure of their belief. If the nocebo hypothesis is true then, regardless of exposure condition (real or sham), IEI-EMF participants should report lower levels of subjective well-being when they judged the base station to be “on” compared to “off”. However, as control participants do not believe they are affected by exposure to EMFs, it was expected that subjective well-being would be the same regardless of participants’ judgments.

## Experiment 1

Experiment 1 aimed to establish whether subjective well-being reported by IEI-EMF and control participants varied as a function of on/off judgments during three different exposure conditions: Global System for Mobile Communication (GSM), Universal Mobile Telecommunications System (UMTS) base station signals, and sham (Eltiti et al., 2007a). It is important to note that the original analyses of the data showed no relationship between exposure condition and well-being in either IEI-EMF or control participants.

In the original study, participants completed four sessions at least 1 week apart comprising one open-provocation and three double-blind sessions (Eltiti et al., 2007a). Only data from the three double-blind sessions were used for the present analyses; one 50-min exposure condition (GSM, UMTS, sham) was administered per double-blind session. Thus, for each participant we had data from two “on” (GSM and UMTS) and one “off” (sham) exposures. During each exposure period, participants completed a range of tasks as well as visual analog scales (VAS) measuring well-being and symptom scales, at regular intervals. Participants also reported whether they thought the base station was “on” or “off”. Note that this judgment was made at the end of each 50-min period to ensure reasonable time for participants to perceive the presence/absence of an EMF signal. In order to assess the effect of judgment on well-being, participants’ judgments were transformed into a participant variable in which only participants that made at least one “on” and one “off” judgment were included in the following analyses. As there were three exposure conditions in which judgments were made (GSM, UMTS, and sham), this allowed for three separate sets of analyses. Specifically, VAS and symptom data by judgment (on vs. off) were compared during sham and GSM, sham and UMTS, and GSM and UMTS exposure conditions in controls and IEI-EMF participants; henceforth these sets of analyses are referred to as sham-GSM, sham-UMTS, and GSM-UMTS, respectively. Exposure order was randomized and counter-balanced across participants. Participants (and researchers) knew that all three exposure conditions would be given, but were blind to the order.

### Materials and Methods

#### Participants

In the original study, 44 IEI-EMF and 114 control participants completed the double-blind portion of the experiment (Eltiti et al., 2007a). Participants were classified as IEI-EMF based on their responses to the Electromagnetic Hypersensitivity Questionnaire (Eltiti et al., 2007b). All IEI-EMF participants explicitly attributed their symptoms to EMF exposure while control participants reported experiencing no symptoms in connection with EMF exposure. The original study received ethical approval from the University of Essex ethics committee and all testing was done in accordance to the Helsinki Convention.

**FIGURE 1:**
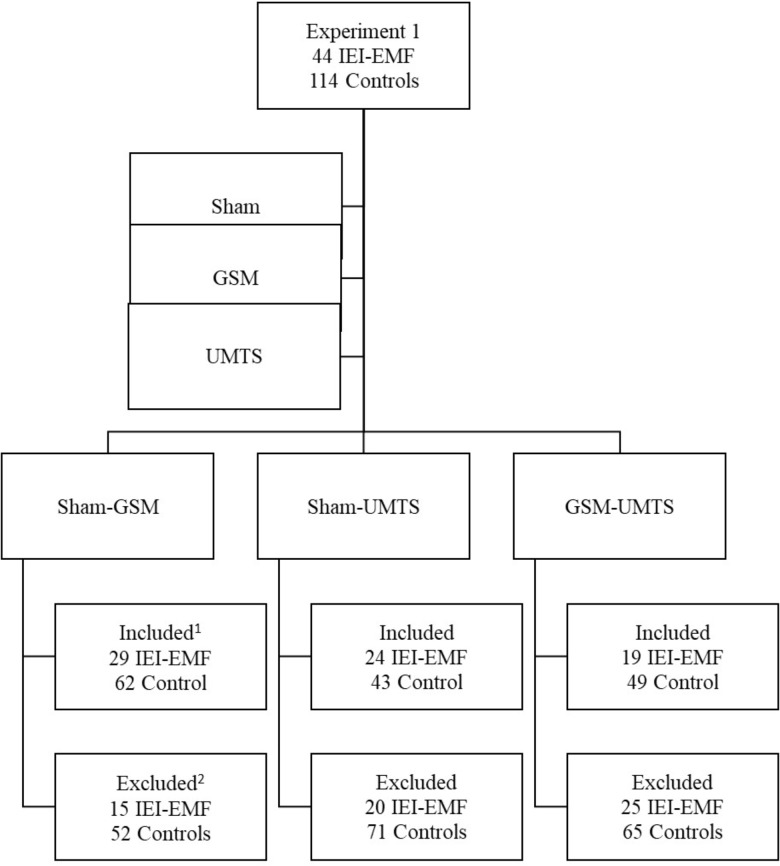
Breakdown of number of IEI-EMF and control participants from Experiment 1 that met the inclusion and exclusion criteria for each set of exposure conditions analyzed. ^1^To be included in the analysis participants had to have made one “on” and one “off” judgment during these exposure conditions. ^2^Participants were excluded from a given analysis if they made either two “on” judgments or two “off” judgments during these exposure conditions as a within-subjects comparison of “on” versus “off” could not be analyzed.

To be eligible for inclusion in the present study’s analysis, participants had to have reported at least one “on” and one “off” judgment. Specifically, participants’ judgments had to either be concordant, that is, judged “on” during real exposure and “off” during sham, or discordant, that is, judged “off” during real exposure and “on” during sham, to be included in the analyses. This criterion was also applied to the GSM-UMTS analysis, participants had to either have judged the GSM condition as “on” and the UMTS condition as “off” or vice-a-versa. Data of participants who judged all sessions to be “on” or all to be “off” were therefore not analyzed as no within-subjects comparisons could be made. It is important to note that the GSM-UMTS analysis served as our control condition where, unlike the sham-GSM and sham-UMTS analyses, a real EMF exposure occurred in both conditions. Hence, any differences emerging between “on” vs. “off” judgments in the GSM-UMTS analysis cannot be imputed to presence or absence of exposure, but is to be uniquely attributed to beliefs. Number of IEI-EMF and control participants who were included and excluded in each analysis is presented in **Figure 1**.

Results from Chi-square analyses showed no significant difference between IEI-EMF and control participants in terms of gender, ethnicity, and chronic illness for any of the three analyses: sham-GSM, sham-UMTS, or GSM-UMTS. **Table 1** provides a complete list of demographics and statistical results. There was also no difference between the groups in terms of marital status for the sham-UMTS analysis; however, there was a significant difference for both the sham-GSM and GSM-UMTS analyses. Independent samples *t*-tests resulted in no significant age difference between IEI-EMF and control participants in the sham-GSM or sham-UMTS analyses, but there was a significant age difference with controls being significantly older than IEI-EMF participants for the GSM-UMTS analysis. Lastly, Chi-square analysis resulted in no significant difference between the IEI-EMF participants and controls in proportion of concordant judgments or order of exposure for any of the three analyses: sham-GSM, sham-UMTS, or GSM-UMTS.

**Table 1:** Participant characteristics for all analyses in Experiment 1.

	Sham-GSM			Sham-UMTS			GSM-UMTS		
Demographics	IEI-EMF (*n* = 29)	Control (*n* = 62)	Statistics^a^	IEI-EMF (*n* = 24)	Control (*n* = 43)	Statistics	IEI-EMF (*n* = 19)	Control (*n* = 49)	Statistics
Age (years)^b^	48.52 (13.51)	50.98 (15.37)	*t*(89) = −0.74, *p* = 0.46	50.08 (12.99)	47.77 (16.33)	*t*(65) = 0.60, *p* = 0.55	41.53 (12.12)	53.53 (14.14)	*t*(66) = −3.26, *p* = 0.002
Gender (% male)	55.2	51.6	χ^2^(1) = 0.10, *p* = 0.75	62.5	58.1	χ^2^(1) = 0.12, *p* = 0.73	47.4	38.8	χ^2^(1) = 0.42, *p* = 0.52
Ethnicity (% White British)	69.0	79.0	χ^2^(9) = 10.46, *p* = 0.31	87.5	74.4	χ^2^(9) = 8.95, *p* = 0.44	68.4	83.7	χ^2^(6) = 8.22, *p* = 0.22
Marital status (%)			χ^2^(5) = 13.90, *p* = 0.016			χ^2^(5) = 7.01, *p* = 0.22			χ^2^(4) = 10.11, *p* = 0.040
Cohabiting	10.3	0.0		12.5	2.3		10.5	2.0	
Divorced	6.9	6.5		8.3	9.3		0.0	4.1	
Married	51.7	69.4		54.2	62.8		42.1	73.5	
Separated	6.9	0.0		4.2	0.0		5.3	20.4	
Single	20.7	24.2		16.7	25.6		42.1	0.0	
Widowed	3.4	0.0		4.2	0.0		0.0	0.0	
Chronic illness (% No)	75.9	67.7	χ^2^(1) = 0.62, *p* = 0.43	62.5	65.1	χ^2^(1) = 0.05, *p* = 0.83	78.9	69.4	χ^2^(1) = 0.62, *p* = 0.43
Judgments (% concordant)	58.6	46.8	χ^2^(1) = 1.11, *p* = .29	58.3	58.1	χ^2^(1) < 0.01, *p* = 0.99	52.6^c^	38.8^c^	χ^2^(1) = 1.08, *p* = 0.30
Exposure order (% Sham – real)	55.2	50.0	χ^2^(1) = 0.21, *p* = 0.65	50.0	48.8	χ^2^(1) < 0.01, *p* = 0.93	42.1^d^	53.1^d^	χ^2^(1) = 0.66, *p* = 0.42

*^a^Chi-square, df, and p-values for all analyzes except age, which was analyzed using independent samples t-tests in which case t-values instead of Chi-square values are reported. ^b^M (SD). ^c^GSM concordant meaning participants judged “on” during the GSM and “off” during the UMTS condition. ^d^GSM – UMTS.*

#### Materials and Apparatus

##### Laboratory and exposure equipment

Testing took place at the Electromagnetic and Health Laboratory, University of Essex. The shielding effectiveness of the testing room was greater than 60 dB for the tested frequencies. The power flux density was nil for sham (no signal) and 10 mW/m^2^ for the GSM and UMTS base station signals. See the original study for a completed description of the laboratory and exposure system (Eltiti et al., 2007a).

##### Subjective well-being

Visual analog scales and symptom scales were utilized to measure subjective well-being. VAS consisted of a 10 cm line with the anchors of not at all and extremely. There were six VAS measuring: anxiety, tension, arousal, relaxation, discomfort, and fatigue. The 57 symptoms from the Electromagnetic Hypersensitivity Questionnaire (Eltiti et al., 2007b) were used to measure both the severity and total number of symptoms experienced.

##### On/off judgment

Upon completion of each 50-min exposure period, participants were asked to report whether they thought the base station had been “on” or “off” and how confident they were of their judgment. If they marked the “on” box, they had to indicate which signal they thought was being emitted: “GSM” or “3G” (i.e., UMTS).

#### Procedures

Prior to testing, all participants signed an informed consent form. During each double-blind session, a 50-min exposure condition was administered during which participants engaged in a 20-min low cognitive load task (they watched the Blue Planet video), a 20-min high cognitive load task (mental arithmetic), two cognitive tests and then reported whether they believed the base station was “on” or “off”. Low and high load tasks were counterbalanced across participants. VAS and symptom scales were completed every 5 min during the low and high load tasks and an additional symptom scale was completed at the end of each session. Each session lasted approximately 1.5 h.

#### Data Analysis

##### Visual analog scales

Prior to analysis, mean scores for the VAS were calculated. Normality of the VAS was analyzed using the Kolmogorov–Smirnov test for normality and by visually inspecting normal and detrended plots. In the sham-GSM analysis, the VAS for discomfort and fatigue were positively skewed; therefore, a square root transformation was used to normalize the data. In the sham-UMTS analysis, just the discomfort VAS was positively skewed and again a square root transformation was used. All other VAS were normally distributed and the data analyses were conducted on the original data.

Mixed factorial ANOVAs with judgment (on, off) as the within-subjects variable and group (IEI-EMF, control) as the between-subjects variable were conducted to determine if beliefs influenced VAS scores. Simple main effects analyses were conducted to directly test our *a priori* hypothesis that IEI-EMF, but not controls, would report poorer levels of well-being during “on” compared to “off” judgments.

##### Symptom scales

Similar to the VAS, mean scores were calculated for the total symptom score (which measured symptom severity) and total number of symptoms. Both the total symptom score and total number of symptoms had a high degree of skewness and kurtosis and did not lend themselves to transformation. Thus, non-parametric statistics were used to analyze the impact of beliefs on symptom data.

Wilcoxon Signed Rank tests were used to determine if there were significant differences in symptoms between on and off judgments for both IEI-EMF and control participants. Additionally, between-group differences in symptoms reported for both on and off judgments were analyzed using Mann–Whitney *U* tests. Lastly, in order to determine if there was an interaction between group and judgment, difference scores (mean on score minus mean off score) were calculated and then Mann–Whitney *U* tests were performed on the difference scores comparing the IEI and control participants. Bonferroni corrections were applied resulting in an alpha of 0.008 for the factorial ANOVAs and Mann–Whitney *U* tests and 0.013 for the Wilcoxon Signed Rank tests. Only results significant at the corrected alpha level are discussed. All data were analyzed using SPSS (v. 22). Copies of all datasets used in these analyses are available from the corresponding author upon reasonable requests.

### Results

#### Visual Analog Scales (VAS)

Means and standard deviations for group by judgment for each VAS as well as exact *F*-values and *p*-values for main effects, interactions, and simple main effect comparisons and partial eta-squared for significant results are presented in **Tables 2**–**4**. **Table 2** contains the results for the sham-GSM analyses, **Table 3** displays the results for the sham-UMTS analyses, and **Table 4** contains the results for the GSM-UMTS analyses.

**Table 2:** Means and standard deviations for each VAS by judgment by group for Sham-GSM exposure conditions in Experiment 1.

	Group								
	IEI-EMF		Control						
	Judgment		Judgment					On vs. Off	
	Off	On	Off	On	Judgment	Group	Judgment × Group	IEI-EMF	Control
VAS	*M* (*SD*)^a^	*M* (*SD*)	*M* (*SD*)	*M* (*SD*)	Statistics^b^	Statistics	Statistics	Statistics	Statistics
Anxiety	1.86 (1.41)	2.63 (1.64)	1.71 (1.19)	1.81 (1.38)	*F* = 8.82, *p* = .004, η^2^ = 0.09	*F* = 3.17, *p* = 0.079	*F* = 5.30, *p* = 0.024, η^2^ = 0.06	*F* = 10.20, *p* = 0.002, η^2^ = 0.10	*F* = 0.35, *p* = 0.56
Tension	1.97 (1.46)	2.78 (1.70)	1.76 (1.21)	1.89 (1.29)	*F* = 11.81, *p* = 0.001, η^2^ = 0.12	*F* = 4.02, *p* = 0.048, η^2^ = 0.04	*F* = 6.03, *p* = 0.016, η^2^ = 0.06	*F* = 12.74, *p* = 0.001, η^2^ = 0.13	*F* = 0.76, *p* = 0.38
Arousal	1.97 (1.50)	2.63 (1.69)	1.61 (1.16)	1.65 (1.15)	*F* = 7.65, *p* = 0.007, η^2^ = 0.08	*F* = 6.44, *p* = 0.013, η^2^ = 0.07	*F* = 6.03, *p* = 0.016, η^2^ = 0.06	*F* = 10.01, *p* = 0.002, η^2^ = 0.10	*F* = 0.08, *p* = 0.78
Relaxation^c^	7.08 (2.03)	6.03 (2.39)	7.42 (1.54)	7.37 (1.57)	*F* = 16.54, *p* < 0.001, η^2^ = 0.16	*F* = 4.90, *p* = 0.029, η^2^ = 0.05	*F* = 13.34, *p* < .001, η^2^ = 0.13	*F* = 21.87, *p* = 0.001, η^2^ = 0.20	*F* = 0.14, *p* = 0.72
Discomfort	1.86 (1.74)	2.74 (1.95)	1.28 (1.24)	1.29 (1.17)	*F* = 8.51, *p* = 0.004, η^2^ = 0.09	*F* = 9.84, *p* = 0.002, η^2^ = 0.10	*F* = 7.60, *p* = 0.007, η^2^ = 0.08	*F* = 11.81, *p* = 0.001, η^2^ = 0.12	*F* = 0.20, *p* = 0.89
Fatigue	2.84 (2.61)	3.19 (2.17)	1.83 (1.60)	1.82 (1.36)	*F* = 2.01, *p* = 0.16	*F* = 9.34, *p* = 0.003, η^2^ = 0.10	*F* = 0.71, *p* = 0.40	*F* = 1.87, *p* = 0.18	*F* = 0.26, *p* = 0.61

*Original untransformed data. ^a^Mean (Standard Deviation). ^b^F- values, P –values, and partial eta-squared. Degrees of freedom for all analyses were 1,89. Partial eta-squared values (indicated as η^2^) are only reported for results with p < 0.05. ^c^High score indicates greater levels of relaxation.*

**Table 3:** Means and standard deviations for each VAS by judgment by group for Sham-UMTS exposure conditions in Experiment 1.

	Group								
	IEI-EMF		Control						
	Judgment		Judgment					On vs. Off	
	Off	On	Off	On	Judgment	Group	Judgment × Group	IEI-EMF	Control
VAS	*M* (*SD*)^a^	*M* (*SD*)	*M* (*SD*)	*M* (*SD*)	Statistics^b^	Statistics	Statistics	Statistics	Statistics
Anxiety	1.86 (1.45)	2.79 (1.75)	1.72 (1.27)	1.71 (1.29)	*F* = 9.22, *p* = 0.003, η^2^ = 0.12	*F* = 3.49, *p* = 0.067	*F* = 9.57, *p* = 0.003, η^2^ = 0.13	*F* = 14.64, *p* < 0.001, η^2^ = 0.18	*F* < 0.01, *p* = 0.96
Tension	2.05 (1.54)	2.97 (1.88)	1.82 (1.25)	1.83 (1.26)	*F* = 9.18, *p* = 0.004, η^2^ = 0.12	*F* = 4.27 *p* = 0.043, η^2^ = 0.06	*F* = 9.06, *p* = 0.004, η^2^ = 0.12	*F* = 14.21, *p* < 0.001, η^2^ = 0.18	*F* < 0.01, *p* = 0.99
Arousal	2.10 (1.57)	2.73 (1.79)	1.70 (1.17)	1.69 (1.27)	*F* = 4.28, *p* = 0.043, η^2^ = 0.06	*F* = 4.90, *p* = 0.030, η^2^ = 0.07	*F* = 4.66, *p* = 0.035, η^2^ = 0.07	*F* = 6.96, *p* = 0.010, η^2^ = 0.10	*F* < 0.01, *p* = 0.94
Relaxation^c^	6.67 (2.51)	6.01 (2.47)	7.42 (1.70)	7.42 (1.72)	*F* = 3.95, *p* = 0.051	*F* = 4.90, *p* = 0.030, η^2^ = 0.07	*F* = 3.95, *p* = 0.051	*F* = 6.15, *p* = 0.016, η^2^ = 0.09	*F* < 0.01, *p* > 0.99
Discomfort	1.78 (1.82)	2.85 (1.95)	1.48 (1.45)	1.47 (1.43)	*F* = 9.94, *p* = 0.002, η^2^ = 0.13	*F* = 4.84, *p* = 0.031, η^2^ = 0.07	*F* = 12.00, *p* = 0.001, η^2^ = 0.16	*F* = 17.06, *p* < 0.001, η^2^ = .21	*F* = 0.07, *p* = 0.80
Fatigue	2.64 (2.39)	3.00 (1.99)	2.24 (1.97)	2.23 (1.74)	*F* = 0.77, *p* = 0.38	*F* = 1.61, *p* = 0.21	*F* = 0.84, *p* = 0.36	*F* = 1.25, *p* = 0.27	*F* < 0.01, *p* = 0.98

*Original untransformed data. ^a^Mean (Standard Deviation) ^b^F- values, P –values, and partial eta-squared. Degrees of freedom for all analyses were 1,65. Partial eta-squared values (indicated as η^2^) are only reported for results with p < 0.05. ^c^High score indicates greater levels of relaxation.*

**Table 4:** Means and standard deviations for each VAS by judgment by group for GSM-UMTS exposure conditions in Experiment 1.

	Group								
	IEI-EMF		Control						
	Judgment		Judgment					On vs. Off	
	Off	On	Off	On	Judgment	Group	Judgment × Group	IEI-EMF	Control
VAS	*M* (*SD*)^a^	*M* (*SD*)	*M* (*SD*)	*M* (*SD*)	Statistics^b^	Statistics	Statistics	Statistics	Statistics
Anxiety	1.94 (1.34)	2.59 (2.07)	1.76 (1.11)	1.79 (1.16)	*F* = 5.27, *p* = 0.025, η^2^ = 0.07	*F* = 2.22, *p* = 0.14	*F* = 4.31, *p* = 0.042, η^2^ = 0.06	*F* = 6.63, *p* = 0.012, η^2^ = 0.09	*F* = 0.04, *p* = 0.84
Tension	2.09 (1.47)	2.69 (2.11)	1.85 (1.15)	1.99 (1.18)	*F* = 4.90, *p* = 0.030, η^2^ = 0.07	*F* = 2.01, *p* = 0.16	*F* = 1.94, *p* = 0.17	*F* = 4.51, *p* = 0.037, η^2^ = 0.06	*F* = 0.60, *p* = 0.44
Arousal	2.02 (1.40)	2.72 (1.95)	1.70 (1.09)	1.77 (1.08)	*F*) = 6.70, *p* = 0.012, η^2^ = 0.09	*F* = 4.15, *p* = 0.046, η^2^ = 0.06	*F* = 4.37, *p* = 0.040, η^2^ = 0.06	*F* = 7.60, *p* = 0.008, η^2^ = 0.10	*F* = 0.22, *p* = 0.64
Relaxation^c^	7.11 (1.68)	6.70 (2.18)	7.12 (1.67)	7.22 (1.45)	*F* = 0.58, *p* = 0.45	*F* = 0.44, *p* = 0.51	*F* = 1.65, *p* = 0.20	*F* = 1.45, *p* = 0.23	*F* = 0.24, *p* = 0.62
Discomfort	1.53 (1.26)	2.41 (1.66)	1.39 (1.35)	1.44 (1.16)	*F* = 8.34, *p* = 0.005, η^2^ = 0.11	*F* = 3.12, *p* = 0.082	*F* = 6.55, *p* = 0.013, η^2^ = 0.09	*F* = 10.30, *p* = 0.002, η^2^ = 0.14	*F* = 0.10, *p* = 0.76
Fatigue	2.29 (1.72)	2.91 (1.93)	1.74 (1.46)	1.88 (1.38)	*F* = 5.31, *p* = 0.024, η^2^ = 0.07	*F* = 4.28, *p* = 0.043, η^2^ = 0.06	*F* = 2.08, *p* = 0.15	*F* = 4.87, *p* = 0.031, η^2^ = 0.07	*F* = 0.67, *p* = 0.42

*Original untransformed data. ^a^Mean (Standard Deviation). ^b^F- values, P –values, and partial eta-squared. Degrees of freedom for all analyses were 1,66. Partial eta-squared values (indicated as η^2^) are only reported for results with p < 0.05. ^c^High score indicates greater levels of relaxation.*

##### Sham-GSM

Separate 2 (judgment: on, off) × 2 (group: IEI-EMF, control) mixed factorial ANOVAs were conducted for each VAS to determine the effect of judgment and group on subjective well-being during the sham and GSM conditions. IEI-EMF participants compared to controls reported higher levels of discomfort and fatigue. Overall, participants reported higher levels of anxiety, tension, arousal, and discomfort, and lower levels of relaxation when they judged the base station to be on compared to off. The group by judgment interaction was significant for relaxation and discomfort. Furthermore, simple main effects analyses revealed that differences in anxiety, tension, arousal, relaxation, and discomfort as a function of on vs. off judgments were significant for IEI-EMF, but not control participants.

##### Sham-UMTS

Separate 2 (judgment: on, off) × 2 (group: IEI-EMF, control) mixed factorial ANOVAs were conducted for each VAS to determine the effect of judgment and group on subjective well-being during the sham and UMTS conditions. Overall, participants reported higher levels of anxiety, tension, and discomfort when they judged the base station to be on compared to off. Furthermore, significant group by judgment interactions were found for anxiety, tension, and discomfort. Simple main effects comparisons revealed that this effect was only significant for IEI-EMF and not control participants.

##### GSM-UMTS

Separate 2 (judgment: on, off) × 2 (group: IEI-EMF, control) mixed factorial ANOVAs were conducted for each VAS to determine the effect of judgment and group on subjective well-being during the GSM and UMTS conditions. The results showed that participants reported higher levels of discomfort when they judged the base station to be on compared to off. None of the group by judgment interactions were significant. However, simple main effects comparisons revealed that IEI-EMF, but not control participants, reported significantly higher levels of arousal and discomfort when they judged the base station to be on compared to off.

#### Symptom Scales

**FIGURE 2:**
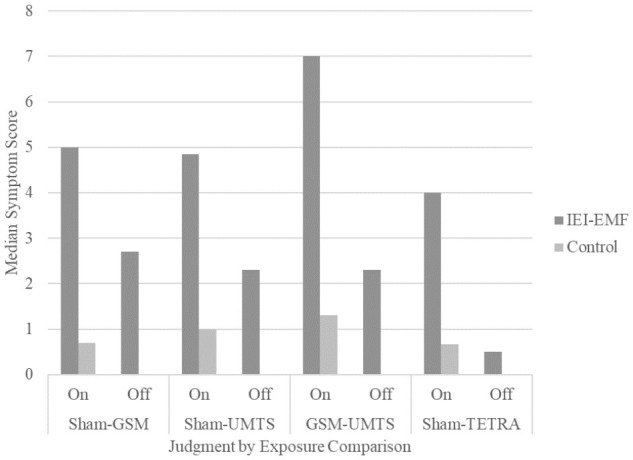
Median total symptom score for IEI-EMF and control participants from Experiments 1 and 2 for both “on” and “off” judgments for each exposure comparison. The median total symptom score for control participants during the “off” judgment for all exposure comparisons were 0.

**FIGURE 3:**
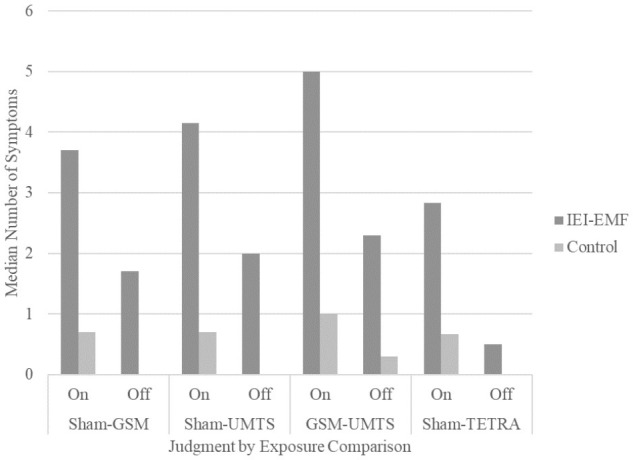
Median total number of symptoms for IEI-EMF and control participants from Experiments 1 and 2 for both “on” and “off” judgments for each exposure comparison. The median total number of symptoms for control participants during the “off” judgment in the Sham-GSM, Sham-UMTS, and Sham-TETRA comparisons were all 0.

**Figure 2** displays the median total symptom score and **Figure 3** the median total number of symptoms for IEI-EMF and control participants by judgment for each exposure comparison set. Exact *p*-values for all analyses are presented in **Table 5**. Wilcoxon Signed Rank tests showed a significant difference between on and off judgments for both IEI-EMF and control participants reporting greater symptom severity and more symptoms when they judged the base station to be on compared to off. Mann–Whitney *U* tests revealed that IEI-EMF participants reported a greater severity of symptoms and total number of symptoms than control participants both during on and off judgments. The above results were the same for the sham-GSM, sham-UMTS, and GSM-UMTS analyses. Additionally, IEI-EMF participants had a larger median difference (on – off) in both severity and total number of symptoms than control participants for the sham-GSM, but not GSM-UMTS analysis. For the sham-UMTS analysis the median difference was significantly larger for IEI-EMF compared to controls in symptom severity, but not in the total number of symptoms.

**Table 5:** Median total symptom scores and total number of symptoms by judgment by group for all analyses in Experiment 1.

	Group										
	IEI-EMF				Control				IEI-EMF vs. Control		
	Judgment			On vs. Off	Judgment			On vs. Off	Off	On	Diff
Sham-GSM	Off	On	Diff^a^	Statistics^b^	Off	On	Diff	Statistics	Statistics	Statistics	Statistics
Total symptom score	2.70	5.00	2.70	*Z* = 3.41, *p* = 0.001	0.00	0.70	0.30	*Z* = 3.41, *p* = 0.001	*Z* = −4.30, *p* < 0.001	*Z* = −5.38, *p* < 0.001	*Z* = −4.05, *p* < 0.001
Total number of symptoms	1.70	3.70	2.00	*Z* = 3.51, *p* < 0.001	0.00	0.70	0.15	*Z* = 3.48, *p* = 0.001	*Z* = −4.22, *p* < 0.001	*Z* = −5.29, *p* < 0.001	*Z* = −3.80, *p* < 0.001
Sham-UMTS											
Total symptom score	2.30	4.85	2.20	*Z* = 2.55, *p* = 0.011	0.00	1.00	0.30	*Z* = 3.48, *p* < 0.001	*Z* = −4.19, *p* < 0.001	*Z* = −4.44, *p* < 0.001	*Z* = −2.62, *p* = 0.009
Total number of symptoms	2.00	4.15	1.65	*Z* = 2.74, *p* = 0.006	0.00	0.70	0.00	*Z* = 2.91, *p* = 0.004	*Z* = −4.09, *p* < 0.001	*Z* = −4.66, *p* < 0.001	*Z* = −2.94, *p* = 0.003
GSM-UMTS											
Total symptom score	2.30	7.00	4.00	*Z* = 2.57, *p* = 0.010	0.00	1.30	0.30	*Z* = 3.31, *p* = 0.001	*Z* = −3.41, *p* < 0.001	*Z* = −3.59, *p* < 0.001	*Z* = −2.31, *p* = 0.021
Total number of symptoms	2.30	5.00	2.70	*Z* = 2.86, *p* = 0.004	0.30	1.00	0.30	*Z* = 3.25, *p* = 0.001	*Z* = −3.71, *p* < 0.001	*Z* = −3.73, *p* < 0.001	*Z* = −2.21, *p* = 0.027

*^a^Difference scores were calculated by subtracting each participant’s mean off score from his or her mean on score. ^b^Z-values and p-values from Mann–Whitney U and Wilcoxon Signed Rank tests.*

### Discussion

The purpose of the present study was to explore the role of belief in determining subjective well-being among IEI-EMF and control participants when they believed they were exposed (or not) to EMFs. The results consistently showed a pattern of IEI-EMF participants reporting lower levels of well-being, both in VAS and symptoms, when they believed they were being exposed to the feared EMFs compared to when they believed they were not. It is important to note that this pattern was still apparent even in the GSM-UMTS analysis in which both conditions contained a real EMF exposure, with the only difference being that participants judged one session to be “on” and the other session to be “off,” thus differences emerging in this analysis can only be attributed to beliefs and not to any discrepancy between presence vs. absence of EMF exposure. Moreover, these differences were stronger than those shown by control participants, indicating that nocebo effects were stronger for IEI-EMF participants. While control participants did not show an effect of belief on reported levels of VAS, there was a significant effect of belief on both the number and severity of symptoms reported. Thus, the presence of actual symptoms may be a more sensitive measure of well-being compared to VAS, which accesses more nuanced changes in global indices of well-being. This result suggests that control participants may also be susceptible to a nocebo effect, albeit to a reduced magnitude than IEI-EMF participants.

## Experiment 2

To further test the hypothesis of a possible nocebo effect, data from our second double-blind provocation study examining a Terrestrial Trunked Radio Telecommunications System (TETRA) signal were analyzed based, once again, on participants’ belief as to whether or not they thought the base station was “on” or “off” (Wallace et al., 2010). Again it is important to note that the original analyses of the data showed no connection between actual EMF exposure and well-being in either IEI-EMF or control participants. Similar to Experiment 1, IEI-EMF and control participants were exposed to both real and sham base station signals in different testing sessions and in each session made a judgment as to whether they believed the base station was “on” or “off”. As hypothesized in Experiment 1, it was predicted that the IEI-EMF participants would report lower levels of well-being when they judged the base station to be “on” compared to “off” regardless of actual exposure. Based on the results of Experiment 1, it was also predicted that control participants might report lower levels of well-being when they judged the base station to be “on” compared to “off” at least in terms of self-reported symptoms.

In the original study, participants completed three sessions at least 1 week apart comprising one open-provocation and two double-blind sessions (Wallace et al., 2010). Only data from the two double-blind sessions were used for the present analyses; thus, one “on” (TETRA) and one “off” (sham) 50-min exposure. Participants (and researchers) were blind to the order of exposure, but knew that both exposure conditions would be administered.

### Materials and Methods

#### Participants

A total of 48 IEI-EMF and 132 control participants completed the double-blind portion of the original study (Wallace et al., 2010). Similar to Experiment 1, all participants completed the Electromagnetic Hypersensitivity Questionnaire (Eltiti et al., 2007b) and those who explicitly attributed experiencing symptoms to exposure to EMFs from mobile phone technology were categorized as IEI-EMF. Control participants reported no symptoms in connection to EMF exposure. Ethical approval for the original study was obtained from University of Essex ethics committee, National Research Ethics Service, and East of England Ambulance Service internal ethics group and all testing was conducted in accordance to the Helsinki Convention.

Participants who judged both exposure conditions to be “on” or both to be “off” were excluded from analysis. **Table 6** shows a complete list of demographics and statistical results for the statistical comparisons made for eligible participants. Most participants were female, white British, and did not have a chronic illness. There were more single than married control participants; however, there were an equal number of single and married IEI-EMF participants. There were no significant differences between the groups in terms of age, gender, ethnicity, marital status, or chronic illness. There was no significant difference between the groups in terms of exposure order (sham followed by TETRA/TETRA followed by sham); however, more control than IEI-EMF participants made concordant judgments.

**Table 6:** Participant characteristics for Experiment 2.

	Sham-TETRA		
Demographics	IEI-EMF (*n* = 34)	Control (*n* = 87)	Statistics^a^
Age (years)^b^	43.47 (14.83)	39.57 (18.37)	*t*(119) = 1.10, *p* = 0.27
Gender (% male)	41.2	47.1	χ^2^(1) = 0.35, *p* = 0.56
Ethnicity (% White British)	67.6	67.8	χ^2^(8) = 3.29, *p* = 0.92
Marital status (%)			χ^2^(5) = 5.62, *p* = 0.35
Cohabiting	14.7	6.9	
Divorced	8.8	6.9	
Married	35.3	37.9	
Separated	2.9	0.0	
Single	35.3	47.1	
Widowed	2.9	1.1	
Chronic illness (% no)	91.2	87.4	χ^2^(1) = 0.35, *p* = 0.56
Judgments (% concordant)	35.3	55.2	χ^2^(1) = 3.86, *p* = 0.049
Exposure Order (% Sham – real)	50.0	48.3	χ^2^(1) = 0.03, *p* = 0.87

*^a^Chi-square, df, and p-values for all analyzes except age, which was analyzed using independent samples t-tests in which case t-values instead of Chi-square values are reported. ^b^M (SD).*

#### Materials and Apparatus

##### Laboratory and exposure equipment

As in Experiment 1, participants were tested at the Electromagnetics and Health Laboratory (University of Essex). The laboratory’s shielding effectiveness was 55–60 dB. The power flux density of the TETRA signal was 10 mW/m^2^ and nil for the sham signal (please refer to the original report for a detailed description; Wallace et al., 2010).

##### Questionnaires

Visual analog scales and symptoms scales were used to measure subjective well-being, identical to Experiment 1. Participants completed a judgment questionnaire for each 50-min exposure period, reporting whether they thought the base station had been “on” or “off” and how sure they were of their judgment.

#### Procedures

The procedures were identical to those used in Experiment 1.

#### Data Analyses

Just as in Experiment 1, mean values were calculated for the VAS and difference scores (mean on minus mean off) were calculated for the total symptom score and total number of symptoms and analyzed using SPSS (v. 22). Normality of the VAS were analyzed using the Kolmogorov–Smirnov test for normality and by visually inspecting normal and detrended plots. These analyses revealed that the VAS, especially for the control group, were skewed. Therefore, square root transformations (and reflect and square root for the relaxation VAS) were conducted resulting in normal distributions. Due to a high degree of kurtosis, neither the mean total symptom score nor mean total number of symptoms lent themselves to transformation; therefore, non-parametric analyses were conducted. The VAS and symptom data were analyzed as in Experiment 1. Datasets are available from the corresponding authors upon reasonable request.

### Results

#### Visual Analog Scales (VAS)

Means and standard errors for each VAS along with exact *p*-values for main effects, interaction, and simple main effects analyses and partial eta-squared for significant results are presented in **Table 7**. Separate 2 (judgment: on, off) × 2 (group: IEI-EMF, control) mixed factorial ANOVAs were conducted for each VAS to determine the effect of judgment and group on subjective well-being. Overall, higher levels of anxiety, tension, arousal, and discomfort and lower level of relaxation were reported when participants judged the base station to be on compared to off with no difference observed for fatigue. Albeit the judgment by group interaction was significant, following the correction for familywise error rate, only for arousal, a similar trend was present for the other VAS (apart from fatigue). Simple main effects analyses revealed that the “on” vs. “off” pattern was only significant for IEI-EMF participants, but not control participants.

**Table 7:** Means and standard deviations for each VAS by judgment by group in Experiment 2.

	Group								
	IEI-EMF		Control						
	Judgment		Judgment					On vs. Off	
	Off	On	Off	On	Judgment	Group	Judgment × Group	IEI-EMF	Control
VAS	*M* (*SD*)^a^	*M* (*SD*)	*M* (*SD*)	*M* (*SD*)	Statistics^b^	*P*(η^2^)	*P*(η^2^)	*P*(η^2^)	*P*(η^2^)
Anxiety	1.49 (1.51)	2.07 (1.82)	1.15 (1.06)	1.28 (1.10)	*F* = 11.80, *p* = 0.001, η^2^ = 0.09	*F* = 3.44, *p* = 0.066	*F* = 3.60, *p* = 0.060	*F* = 9.89, *p* = 0.002, η^2^ = 0.08	*F* = 2.11, *p* = 0.15
Tension	1.58 (1.50)	2.18 (1.95)	1.21 (1.08)	1.35 (1.19)	*F* = 9.85, *p* = 0.002, η^2^ = 0.08	*F* = 3.35, *p* = 0.070	*F* = 3.23, *p* = 0.075	*F* = 8.47, *p* = 0.004, η^2^ = 0.07	*F* = 1.60, *p* = 0.21
Arousal	1.51 (1.47)	2.38 (1.94)	1.23 (1.15)	1.36 (1.35)	*F* = 25.02, *p* < 0.001, η^2^ = 0.17	*F* = 5.02, *p* = 0.027, η^2^ = 0.04	*F* = 13.01, *p* < 0.001, η^2^ = 0.10	*F* = 25.77, *p* < 0.001, η^2^ = 0.18	*F* = 1.73, *p* = 0.19
Relaxation^c^	6.53 (1.73)	5.85 (1.73)	6.79 (1.42)	6.75 (1.48)	*F* = 8.28, *p* = 0.005, η^2^ = 0.07	*F* = 3.25, *p* = 0.074	*F* = 6.32, *p* = 0.013, η^2^ = 0.05	*F* = 10.11, *p* = 0.002, η^2^ = 0.08	*F* = 0.12, *p* = 0.73
Discomfort	1.72 (1.86)	2.02 (1.57)	1.15 (1.13)	1.27 (1.25)	*F* = 9.61, *p* = 0.002, η^2^ = 0.08	*F =* 4.36, *p* = 0.039, η^2^ = 0.04	*F* = 2.78, *p* = 0.098	*F* = 7.91, *p* = 0.006, η^2^ = 0.06	*F* = 1.82, *p* = 0.18
Fatigue	2.28 (1.93)	2.46 (1.78)	1.61 (1.53)	1.73 (1.64)	*F* = 2.12, *p* = 0.148	*F* = 4.73, *p* = 0.032, η^2^ = 0.04	*F* = 0.61, *p* = 0.44	*F* = 1.74, *p* = 0.19	*F* = 0.41, *p* = 0.53

*Original untransformed data. ^a^Mean (Standard Deviation). ^b^F- values, P -values, and partial eta-squared. Degrees of freedom for all analyses were 1,119. Partial eta-squared values (indicated as η^2^) are only reported for results with p < 0.05. ^c^High score indicates greater levels of relaxation.*

#### Symptoms Scales

Wilcoxon Signed Rank tests showed that there was a significant effect of judgment with both IEI-EMF and control participants reporting a greater severity of symptoms and more symptoms when they judged the base station to be “on” compared to “off”. Mann–Whitney *U* tests also revealed that IEI-EMF participants consistently reported a greater severity of symptoms and more symptoms compared to controls; regardless of the base station being judged as on or off. Analyses of the difference scores (on–off) revealed that the median difference in symptom severity, but not total number of symptoms (albeit the trend was comparable), was significantly greater for IEI-EMF compared to control participants. **Figure 2** displays the median total symptom score and **Figure 3** the median total number of symptoms for IEI-EMF and control participants by judgment for each exposure comparison set. Exact *p*-values for all analyses are presented in **Table 8**.

**Table 8:** Median total symptom scores and total number of symptoms by judgment by group in Experiment 2.

	Group										
	IEI-EMF				Control				IEI-EMF vs. Control		
	Judgment			On vs. Off	Judgment			On vs. Off	Off	On	Diff
	Off	On	Diff^a^	Statistics^b^	Off	On	Diff	Statistics	Statistics	Statistics	Statistics
Total symptom score	0.50	4.00	1.83	*Z* = 4.00, *p* < 0.001	0.00	0.67	0.33	*Z* = 3.82, *p* < 0.001	*Z* = −2.85, *p* = 0.004	*Z* = −4.65, *p* < 0.001	*Z* = −3.27, *p* = 0.001
Total number of symptoms	0.50	2.83	1.50	*Z* = 3.28, *p* = 0.001	0.00	0.67	0.33	*Z* = 4.29, *p* < 0.001	*Z* = −2.97, *p* = 0.003	*Z* = −4.80, *p* < 0.001	*Z* = −2.31, *p* = 0.021

*^a^Difference scores were calculated by subtracting each participant’s mean off score from his or her mean on score. ^b^Z-values and p-values from Mann–Whitney U and Wilcoxon Signed Rank tests.*

## General Discussion

The present studies explored whether a nocebo effect could explain the presence of symptoms in IEI-EMF individuals in two provocation studies. It was hypothesized that regardless of actual exposure, IEI-EMF individuals would report lower levels of well-being when they judged the base station to be “on” compared to “off”. This hypothesis was supported in that IEI-EMF individuals in Experiment 1 consistently reported (in all analyses) higher levels of discomfort, a greater number of symptoms, and severity of symptoms when they judged the base station to be “on” compared to “off”. Additionally, higher levels of anxiety (sham-GSM, sham-UMTS), tension (sham-GSM, sham-UMTS), arousal (sham-GSM, GSM-UMTS) and lower levels of relaxation (sham-GSM) were reported during “on” compared to “off” judgments. Likewise in Experiment 2, IEI-EMF individuals reported higher levels of anxiety, tension, arousal, and discomfort; lower levels of relaxation; a greater number of symptoms; and severity of symptoms when they judged the base station to be “on” compared to “off”. While in Experiment 2 the group by judgment interactions were not always significant at the more stringent alpha level, the consistent pattern of the findings obtained in both experiments strengthens the conclusion that the presence of symptoms in IEI-EMF individuals is primarily driven by their belief that exposure to EMFs will cause negative health effects rather than the actual exposure itself.

Interestingly, in both Experiments 1 and 2 control participants also reported more symptoms and greater symptom severity when they too judged the base station to be “on” (albeit to a significantly lesser extent than IEI-EMF individuals). This was contrary to our initial prediction as control participants had been selected based on their self-report that they did not experience any negative health effect due to EMF exposure. However, studies have shown that non-IEI-EMF individuals can be susceptible to an EMF-induced nocebo effect (Schweiger and Parducci, 1981; Witthöft and Rubin, 2013). Furthermore, research has demonstrated that the nocebo effect can occur with verbal suggestion alone without any previous experience or conditioning (Colloca et al., 2008). Simply listing symptoms in the consent form as possible side effects can elicit a nocebo effect for those particular symptoms (Myers et al., 1987). Participants in the present studies were fully informed that we were investigating possible negative health effects due to EMF exposure. Also, during this time there was widespread media interest in IEI-EMF and the studies we were conducting (Eldridge-Thomas and Rubin, 2013). Thus, it is possible that although these control participants would not normally explicitly attribute their symptoms to exposure to EMFs, in the context of the study they may have judged the base station to be “on” if they experienced any symptoms during the course of the experiment.

The above findings are similar to those of previous studies that showed a nocebo effect in the context of perceived EMF exposure (Schweiger and Parducci, 1981; Landgrebe et al., 2008; Szemerszky et al., 2010; Witthöft and Rubin, 2013). One key difference between the present study and previous research is the comparison of “on” vs. “off” judgments. In previous research, participants’ symptoms were only measured under conditions in which they were lead to believe that they were being exposed to EMFs and either that that particular EMF exposure was associated with a specific set of symptoms (Schweiger and Parducci, 1981; Szemerszky et al., 2010; Witthöft and Rubin, 2013) or utilized IEI-EMF participants (Landgrebe et al., 2008). The findings of the present study strengthens those of previous research by demonstrating that *post hoc* treatment guesses of whether an EMF device is “on” or “off” are linked to symptom severity and well-being.

It is important to note the role expectations play in the presence of a nocebo response; it is the individual’s expectation that a particular negative outcome will occur that in essence causes that negative outcome (Hahn, 1997; Colloca and Miller, 2011). The mechanism by which a nocebo response may occur is possibly due to a negative expectation causing the individual to focus more inwardly on specific physiological outcomes thereby lowering the threshold necessary to experience them (Hahn, 1999). In the case of IEI-EMF, when an IEI-EMF individual perceives that he or she is being exposed to EMFs it is the expectation that EMFs are harmful that then leads the individual to experience negative health effects (Dieudonńe, 2016). For example, if an individual associates pain in the head with EMF exposure from a mobile phone, this expectation may cause the individual to attend more to pain perception thus lowering his or her pain threshold when in the presence of a mobile phone.

In previous studies, expectations were directly manipulated by providing participants information that would lead them to expect specific negative health outcomes (Schweiger and Parducci, 1981; Szemerszky et al., 2010; Witthöft and Rubin, 2013) or by using IEI-EMF participants who already held negative expectations concerning EMF exposure (Landgrebe et al., 2008). Interestingly, Szemerszky et al. (2010) reported that participants who scored higher on an IEI-EMFs scale reported greater expectations that they would experience symptoms when exposed to EMFs and reported greater symptom severity in response to a sham EMF exposure. All IEI-EMF participants in our study endorsed and could articulate that they experienced specific symptoms that they strongly believed were due to EMF exposure from mobile telephone devices. Thus, it is reasonable to assume that the IEI-EMF participants in our experiments had negative expectations concerning EMF exposure. In addition, participants in our studies knew that in Experiment 1 two of the three double-blind sessions and in Experiment 2 one of the two double-blind sessions would contain a real EMF exposure. The expectation that they would indeed be exposed to EMFs along with IEI-EMFs strongly held belief that exposure to EMFs causes negative health effects can explain the presence of symptoms in these experiments.

By examining the data from our two previous studies (Eltiti et al., 2007a; Wallace et al., 2010) based on participants’ belief, we were able to demonstrate that a nocebo effect provides a reasonable explanation for the presence of symptoms in IEI-EMF individuals. Some may argue that rather than participants’ belief that the base station was “on” eliciting their symptoms, it was the presence of symptoms that led them to judge the base station to be “on”. The present study is limited as it is, of course, a *post hoc* analysis of our original data and this explanation cannot entirely be ruled out and may very well be the case for control participants who on average did report 0 symptoms when they judged the base station to be “off”. Importantly, IEI-EMF participants were not symptom-free when they reported the base station to be “off,” but rather had significantly fewer symptoms. Thus, at least for IEI-EMF participants, the mere presence of symptoms did not automatically results in an “on” judgment, but rather these symptoms would have had to reach a certain threshold before judging the base station to be “on”. The more parsimonious explanation is that it was IEI-EMFs’ pre-existing belief that EMFs are harmful that triggered their symptoms rather than that their symptoms led them to believe that the base station was on. Either way, the results clearly show that the symptoms were not caused by EMF exposure and that when the symptoms reached some threshold they were then attributed to EMFs rather than some other environmental or biological factor, which in itself is an interesting finding that deserves further exploration.

Future research should continue to identify factors that can lead to nocebo beliefs, how these beliefs are formed and maintained and methods for counteracting these beliefs once they become established in the mind. Very little research has actually focused on treatments for IEI-EMF individuals; however, preliminary findings have suggested that cognitive behavioral therapy (CBT) may be an effective form of treatment (Rubin G.J. et al., 2006). This fits in well with current findings from meta-analyse that have shown CBT as efficacious in treating medically unexplained physical symptoms, such as chronic fatigue syndrome (Malouff et al., 2008), fibromyalgia (Glombiewski et al., 2010), and irritable bowel syndrome (Li et al., 2014). A helpful approach to treating individuals with medically unexplained physical symptoms was outlined by Smith et al. (2003) in which, alongside building patient rapport, CBT plays an important role in helping patients restructure their illness perceptions and beliefs regarding symptom causation. More recently Van den Bergh et al. (2017) have proposed a treatment strategy for IEI illnesses that focuses on modifying symptom perception and expectations. Even so, much more research is needed in this area especially for medically unexplained physical symptoms associated with IEI-EMF, multiple chemical sensitivity, sick building syndrome, and infrasound hypersensitivity in which the vast majority of research has focused on identifying causal factors and very little research has actually examined the effectiveness of various treatments for these illnesses.

## Author Contributions

All authors contributed to the design and interpretation of the results of this study. SE and DW collected the initial data. SE analyzed the data and wrote the manuscript. DW, RR, and EF contributed to the interpretation of the data and provided critical intellectual revision of the manuscript. All authors approved the manuscript and agreed to be accountable for all aspects of the work.

## Conflict of Interest Statement

SE served as an expert witness in a civil case involving Wi-Fi in a private school. The remaining authors declare that the research was conducted in the absence of any commercial or financial relationships that could be construed as a potential conflict of interest. The reviewer SH and handling Editor declared their shared affiliation.
